# Application of dynamic enhanced scanning with GD-EOB-DTPA MRI based on deep learning algorithm for lesion diagnosis in liver cancer patients

**DOI:** 10.3389/fonc.2024.1423549

**Published:** 2025-01-06

**Authors:** Bo Liu, Jinhua Yang, Yifei Wu, Xi Chen, Xueru Wu

**Affiliations:** Department of Radiology, Ordos Central Hospital, Ordos, Inner Mongolia, China

**Keywords:** liver lesion, detection, and classification, Gd-EOB-DTPA, deep learning, magnetic resonance

## Abstract

**Background:**

Improvements in the clinical diagnostic use of magnetic resonance imaging (MRI) for the identification of liver disorders have been made possible by gadolinium ethoxybenzyl diethylenetriamine pentaacetic acid (Gd-EOB-DTPA). Gd-EOB-DTPA-enhanced magnetic resonance imaging (MRI) technology is in high demand.

**Objectives:**

The purpose of the study is to segment the liver using an enhanced multi-gradient deep convolution neural network (EMGDCNN) and to identify and categorize a localized liver lesion using a Gd-EOB-DTPA-enhanced MRI.

**Methods:**

We provided the classifier images of the liver in five states (unenhanced, arterial, portal venous, equilibrium, and hepatobiliary) and labeled them with localized liver diseases (hepatocellular carcinoma, metastasis, hemangiomas, cysts, and scarring). The Shanghai Public Health Clinical Center ethics committee recruited 132 participants between August 2021 and February 2022. Fisher’s exact test analyses liver lesion Gd-EOB-DTPA-enhanced MRI data.

**Results:**

Our method could identify and classify liver lesions at the same time. On average, 25 false positives and 0.6 real positives were found in the test instances. The percentage of correct answers was 0.790. AUC, sensitivity, and specificity evaluate the procedure. Our technique outperforms others in extensive testing.

**Conclusion:**

EMGDCNN may identify and categorize a localized hepatic lesion in Gd-EOB-DTPA-enhanced MRI. We found that one network can detect and classify. Radiologists need higher detection capability.

## 1 Introduction

To create an MRI contrast agent that is specific to the bile and liver, Gd-DTPA has its molecular structure adjusted by including the fat-soluble ethoxybenzyl group (EOB). Gd-EOB-DTPA has exceptional dynamic boosting powers of MRI contrast agents and also has unique biological features (1). Tissue responses to a reduced T1 relaxation time may be compared to the dynamic augmentation produced by Gd-multi-phase DTPA. Because of the presence of natural anion shipping polypeptide 1B3 (OATP1B3) on the cell layer of the hepatic dissemination sinus, the majority of the difference that is given intravenously with Gd-EOB-DTPA is absorbed by normal liver cells within ten to twenty minutes. Subsequently, the drug is eliminated by MRP2 in the biliary membrane of the liver. In (2), this era is called the hepatobiliary epoch. As an example, the contrast agent Gd-DTPA may be excreted on a trip to the restroom. When the kidneys and liver aren’t cutting it, this freedom feature might take over (3). In contrast, hypointensity signal is seen in T1WI images from the hepatobiliary explicit period because Hepatocellular Carcinoma (HCC) cells cannot explicitly take in Gd-EOB-DTPA due to their low OATP1B3 articulation. The weakened signal of cirrhotic nodules makes them stand out in contrast to the healthy liver tissue around them. The rate of intrahepatic retention of Gd-EOB-DTPA is a measure of liver cell health (4). Gd-EOB-DTPA is administered intravenously and fully eliminated the next day. These features allow for the evaluation of liver cell movement via attractive reverberation imaging (X-ray) with the hepatobiliary-explicit differentiation specialist Gd-EOB-DTPA, specifically by observing the sign shifts of injuries over the direction of the hepatobiliary stage (5) and the blood supply example of HCC wounds with dynamic improved filtering.

Multiple phases of dynamic amplification, manifesting as “quick in and wash out,” describe the vast majority of tiny HCC lesions. We refer to tumors like this one as “tiny HCC with a strong blood supply.” To make a quick diagnosis, all you need to do is show a weak signal during the liver-gallbladder-specific phase (6). It may be challenging to establish the standard qualitative diagnosis using enhanced MRI in cases of inadequate blood flow due to atypical symptoms and intrahepatic localized perfusion anomalies. Based on the most recent version of the Liver Imaging Report and Information Framework (7), hepatic hypovascular sores are defined as hepatic lesions with blood vessel improvement that is not precisely or comparable to that of normal liver parenchyma (LI-RADS). Preoperative diagnosis of these ulcers is challenging. In this case, the use of Gd-EOB-DTPA in conjunction with X-ray might lend credence to anecdotal evidence and perhaps establish the presence of a minimal HCC. Throughout the hepatobiliary phase, MRI that has been enhanced with Gd-EOB-DTPA is quite useful. The unique nature of hepatocytes results in poor absorption, hence hepatocellular carcinomas often have a modest signal (8). American and international researchers have recently focused on developing better methods for detecting and diagnosing liver cancer at an early stage (9). Gd(EOB)- DTPA-enhanced X-ray was shown to be the most reliable in a head-to-head comparison with plain X-ray, dynamic improvement multi-cut winding CT, and the gold standard extracellular space contrast specialist Gd (DTPA). In Europe (2004), the United States (2008), and Japan (2015), Gd-EOB-DTPA-enhanced attractive reverberation imaging (X-ray) is the gold standard for identifying and organizing liver illness (2010). (2015). (2010). This method has been shown to increase diagnosis accuracy for HBV-related small liver carcinoma.

Hepatic X-ray imaging using Gd-EOB-enhanced DTPA is anticipated. When it comes to differentiating between HCCs with a rich or lacking blood supply, the Gd-EOB-DTPA-improved X-ray has a more noticeable identification responsiveness than dynamic differentiation improved CT and Gd-DTPA-improved X-ray (10). Compared to other imaging modalities, X-ray improved with Gd-EOB-DTPA has been proven to have much higher detection rates and analytical accuracy for early stage liver disease. Although standard enhanced X-ray has shown to be an effective tool for identifying HCC in growths narrower than 2.0 cm (11), it is not yet at pace with other methods in terms of accuracy and subtlety. The study discovered that Gd-EOB-DTPA-enhanced X-ray may detect liver tumors 2 cm in size with a sensitivity of 0.92 and an explicitness of 0.95 (12). When comparing X-ray enhancement techniques for the detection of small HCCs, Gd-EOB-DTPA may be more sensitive than MSCT multi-stage dynamic upgraded filter, X-ray plain output, and dynamic regular difference (width 2.0 cm). With its benefits over competing methods, Gd-EOB-DTPA may identify liver cancer at its early stages (13). Because of this, several scientists have proposed that integrating hepatobiliary stage imaging with other approaches might improve the diagnostic accuracy and sensitivity of HCC (2 cm). Gd-EOB-enhancement DTPAs not only mimic the action of regular contrast agents but also boost the visibility of cirrotic nodules and tiny hepatocellular carcinoma (HCC) present during the hepatobiliary stage.

Some forms of early liver tumors have been demonstrated in similar trials to exhibit an unusual vascular enhancement pattern, with either no strengthening at all or very little strengthening. These tumors may be evolving from high-grade dysplastic nodules (HGDN) into a more aggressive type of hepatocellular carcinoma. This decrease in portal venous blood flow in early-stage liver cancer suggests that the arterial blood supply is not the primary source of oxygen and nutrients. Consequently, the MRI lesion may seem abnormal when enhanced with Gd-EOB-DTPA. The decreased recurrence of sores communicating natural anion shipping polypeptide 8 (OATP8) (14) demonstrates hepatobiliary hypersensitivity due to the specialized uptake of differentiation specialists by liver cells. It is an attempt to tell high-risk knobs, which may develop into rich blood supply sores on imaging, apart from early HCC. Six percent to fifteen percent of small HCCs had a distinctive boosting mechanism during the hepatobiliary-specific phase. Not only did it include focal nodular hyperplasia (FNH) and FNH-like nodules, but it also covered the phases of small-cell liver cancer that are unique to the liver. Distinguishing is difficult using standard imaging methods. Specifically, Gd-EOB-DTPA-enhanced X-rays may aid in the detection of incredible, tiny knobs of HCC and the differentiation of these from FNH-like lesions. Introspective research was conducted on twenty patients with abnormal nodular HCC and twenty-one patients with FNH-like knobs; during the hepatobiliary stage, the scientists observed an improvement grouping of HCC knobs similar to that of FNH-like knobs; however, a significant wash impact was seen during the entry venous step and the momentary stage (P 0.0001). Distinguishing aberrant HCC knobs has a 90% responsiveness and a 100% explicitness. Time wasting was the most important variable substantially linked to HCC in a multivariate strategic relapse analysis (odds ratio, 7.019; P = 0.042). This study found that patients with aberrant knobs benefit more from Gd-EOB-DTPA-enhanced X-ray for the diagnosis of HCC than those who received regular Gd-DTPA-enhanced X-ray (15).

Although there is a strong correlation between a high AFP level and a bad prognosis, the pace at which AFP levels rise varies widely from person to person. In certain HCC patients with elevated AFP, enhanced CT and normal MRI may overlook microtumor lesions. On conventional MRI, lesions of early-stage small HCC appeared isointense and hypointense on the T1WI, and somewhat hyperintense on the T2WI, although the arterial phase enhancement was not easily discernible. The field has not seen a large output of research from either domestic or international scholars. Therefore, this has to be confirmed by other research. It has been shown that the levels of AFP in some individuals with hepatocellular carcinoma are minimal, whereas in others they are quite high (16). Slightly hyper-focused arterial perfusion problems can only be seen by enhanced CT or ordinary enhanced MRI. Gd-EOB-DTPA-enhanced MRI, on the other hand, has the potential to improve the identification rate of minor lesions by displaying the typical imaging characteristic of hypointensity in the hepatobiliary phase. Unfortunately, many people don’t discover they have hepatocellular carcinoma (HCC), a particularly deadly form of liver cancer, until it’s too late. Surgical removal of the liver is the most common therapy. The prognosis for most persons with HCC is dismal even after extensive surgery due to the resiliency of the illness. Some individuals may develop intra-hepatic metastases before surgery, which are notoriously hard to identify and are often overlooked. “Preoperative detection of intra-hepatic metastases is strongly connected with the Barcelona staging of HCC, the development of surgical methods, and patient outcome (17). The patient’s Barcelona staging before to surgery might be affected by this. As a result, minimizing tumor recurrence and increasing overall survival may depend on the ability to identify tiny intra-hepatic lesions before to surgery and to precisely locate small lesions to be excised after surgery. Despite enhanced CT being the standard for radiologic diagnosis of HCC, it is frequently unable to identify even the smallest liver lesions. When it comes to identifying subtle tumors within the liver, DCE-MRI outperforms improved CT. The use of liver-specific contrast agents has been linked to an improvement in the presentation of localized liver lesions, especially those with a width of less than 1 cm (18). This class of drugs is effective throughout the hepato-biliary phase because they increase signs of hepatic parenchymal enhancement. The study’s author showed that individuals with malignant liver cirrhosis have low diagnostic signals in the delayed phase, while patients with benign lesions have signals that are uniformly distributed or are strong.

When it comes to detecting liver abnormalities, differential contrast-enhanced MRI (DCE-MRI) may be more effective than enhanced multi-detector computed tomography (MDCT). Researchers have shown that DCE-MRI outperforms MDCT in terms of sensitivity, specificity, and diagnostic accuracy when it comes to detecting HCC and metastasized malignancies. DCE-X-ray (which incorporates further filtering of hepatocyte stages) has been demonstrated to detect aberrant high-grade dysplastic knobs (HGDN) and early stage HCC with more sensitivity than conventionally enhanced X-ray (19). A brand-new liver-specific expert in the art of differentiating Gd-EOB-DTPA has several particularly appealing properties for experts in the field of extracellular difference (dynamic stage) (static stage). Gd-EOB-DTPA is excreted through the biliary and renal systems, and it has been reported to enhance both the detection and characterization of wounds. When comparing X-ray with Gd-EOB-DTPA to MDCT and X-ray to other difference experts, X-ray with Gd-EOB-DTPA is the plausible winner in identifying and diagnosing liver abnormalities. When a liver abnormality cannot be consistently identified by CT, enhanced MRI is the recommended diagnostic method. It’s unclear (20) whether those who have been diagnosed with HCC using updated CT also need detection by upgraded X-ray. To determine whether X-ray is necessary for patients with HCC authorized by MDCT, we compared the symptomatic performance of Gd-EOB-DTPA dynamic differentiation upgraded X-ray and enhanced 64-cut CT in detecting intrahepatic sores in HCC patients.

## 2 Method and methodology

### 2.1 Proposed methodology

To evaluate the liver lesion, the Gd-EOB-DTPA-enhanced MRI findings are analyzed using Fisher’s exact test.

SPSS was used to perform statistical analysis. To illustrate the radiomic signature’s capacity for prediction, receiver operator characteristic curves were built. Fisher’s exact probability approach was used to evaluate its importance. Prognostic evaluations were made using Kaplan-Meier analyses. The cutoff for significance was P < 0.05. In statistical hypothesis testing, the p-value quantifies evidence against the null hypothesis, representing the likelihood of observing the test statistic or more extreme values assuming the null is true. If the p-value falls below a predetermined significance level, usually α=0.05, the null hypothesis is rejected in favor of the alternative, indicating statistical significance. For instance, a p-value of 0.0001 suggests strong evidence against the null hypothesis at the 0.05 significance level.

To clarify, the p-value is a measure that helps researchers determine whether the results of their study are statistically significant. When conducting a statistical test, a p-value less than 0.05 is typically considered the threshold for statistical significance. This means that if the p-value is less than 0.05, there is less than a 5% probability that the observed data (or something more extreme) would occur by random chance alone, assuming the null hypothesis is true. Consequently, a p-value below this threshold provides sufficient evidence to reject the null hypothesis and conclude that the observed effect or difference is statistically significant. Conversely, if the p-value is 0.05 or higher, the result is not deemed statistically significant, and the null hypothesis cannot be rejected based on the data.

**Figure 1:**
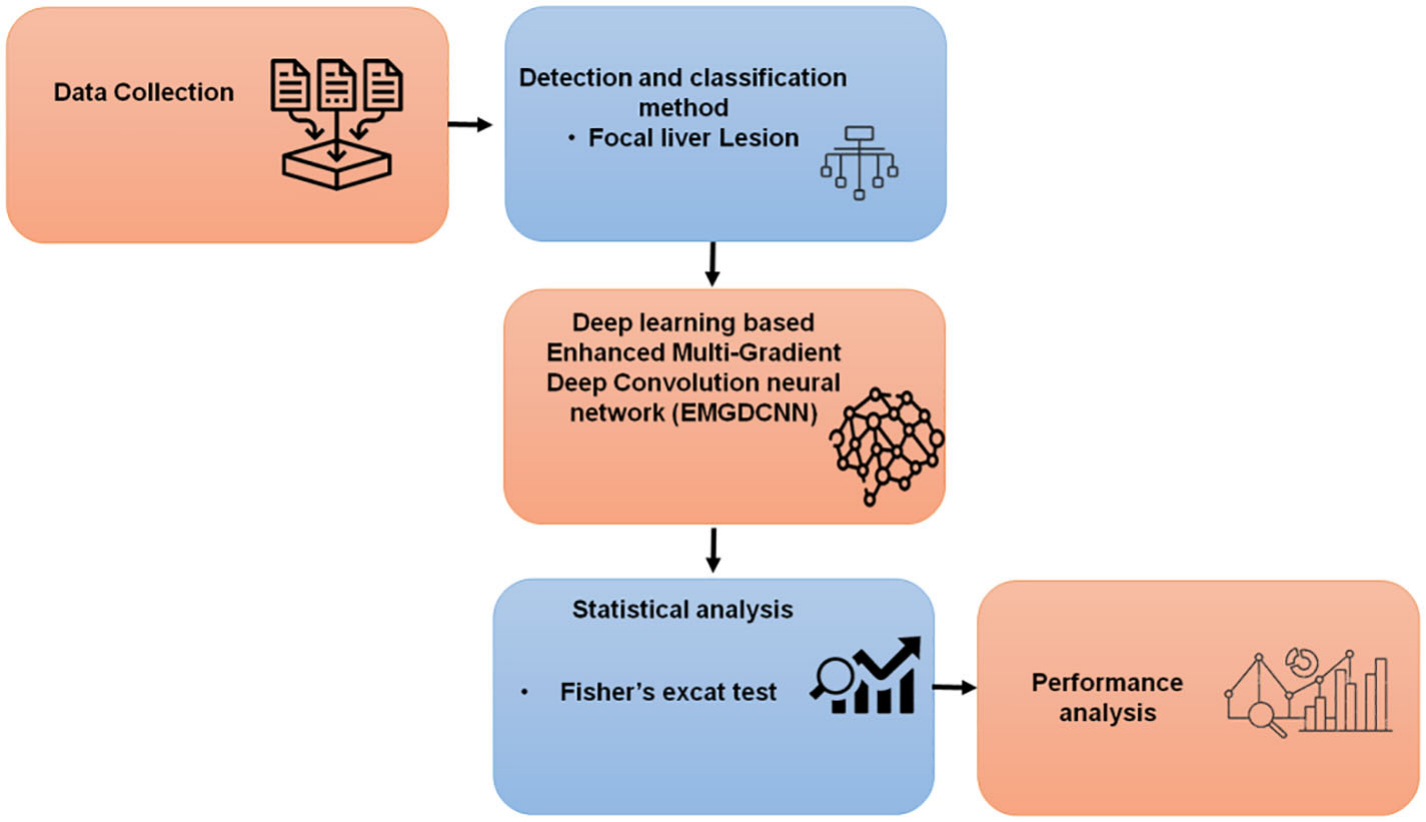
Flowchart of the methodology.

A liver-specific T1 contrast agent for magnetic resonance imaging is gadolinium ethoxybenzyl diethylenetriamine Penta acetic acid (Gd-EOB-DTPA) (MRI). Gd-EOB-DTPA-enhanced MRI has been shown in several studies to have superior diagnostic accuracy than other imaging modalities. When it comes to detecting tiny colorectal liver metastases in individuals that have medium to severe inflammatory infiltration of the liver, Gd-EOB-DTPA-enhanced MRI performs significantly better than second-generation MDCT. Gd (EOB)-DTPA-enhanced MRI creates a high quantity of images using a technique that consists of five steps. Radiologists have a time-consuming process to go through to discover and diagnose lesions in Gd-EOB-DTPA-enhanced MR images. The suggested procedure’s flowchart is shown in **Figure 1**.

### 2.2 Dataset

#### 2.2.1 Research objects

The study was authorized by the ethics committee of the Shanghai Public Health Clinical Center (21). Between August 2021 and February 2022, a total of 132 individuals were recruited, with the majority having chronic hepatitis B and a Child-Pugh score of less than 7. These participants were categorized into four groups based on their Scheuer-Ludwig score (L): L1 (n = 30), L2 (n = 28), L3 (n = 32), and L4 (n = 42) for liver aspiration biopsies (shown as **Table 1**). The clinical diagnosis of liver lesions using gadolinium ethoxybenzyl diethylenetriamine pentaacetic acid (Gd-EOB-DTPA)-enhanced MRI demonstrated high efficacy. For the analysis, labeled images of localized liver lesions were used as output, with a five-phase series serving as input.

**Table 1:** Patient characteristics upon enrollment (Dataset).

	Overall		significant			Advanced			Cirrhosis	
Sex										
**Men**	92(70.5%)		79 (77.5%)			62 (82.4%)			37 (85.7%)	
**women**	39(29.6%)		24 (22.6%)			14 (17.6%)			6 (14.3%)	
**Age(years)**	45.8 ± 13.2		47.7 ± 13.3			51.8 ± 12.6			52.6 ± 11.1	
Fibrosis score										
**L1**	30 (22.7%)		0 (0%)			0 (0%)			0 (0%)	
**L2**	28 (21.2%)		29 (27.45%)			0 (0%)			0 (0%)	
**L3**	32(24.2%)		33 (31.4%)			34 (43.2%)			0 (0%)	
**L4**	42 (31.8%)		43 (41.2%)			44 (56.8%)			44 (100%)	
**Group**		+		–	+		–	+		–
**Test**		31		9	22		18	13		27
**Training**		71		21	52		40	29		63

Overall: This column represents the total dataset characteristics without stratification. Significant: Data for patients categorized as having significant liver fibrosis but not yet classified as advanced or cirrhosis. Advanced: Data for patients with advanced liver disease, characterized by more severe fibrosis but not necessarily cirrhosis. Cirrhosis: Data for patients diagnosed with cirrhosis, representing the most advanced stage of liver disease. Sex: Distribution of male and female participants within each category. Men: Number and percentage of male participants. Women: Number and percentage of female participants. Age (years): Mean age ± standard deviation of participants within each category. Fibrosis score: Distribution of participants according to their Scheuer-Ludwig fibrosis scores (L1, L2, L3, L4), where respectively indicates: Mild fibrosis; Moderate fibrosis; Severe fibrosis; Cirrhosis. Group: Indicates how the dataset was divided into test and training sets for model evaluation. Test: Number of cases allocated to the test set. Training: Number of cases allocated to the training set.

##### Inclusion criteria

1. Diagnostic evaluation of severe hepatitis B.
2. No history of claustrophobia, chronic renal disease, or conditions contraindicating MRI.
3. Age of 18 years or older.
4. Daily alcohol intake of no more than 20 grams.
5. Signed consent forms indicating informed participation.

##### Exclusion criteria

1. The presence of other liver diseases.
2. Child-Pugh score greater than or equal to 7 (indicating moderate to severe liver dysfunction).
3. Prior treatment with antiviral, antifibrotic, or anti-inflammatory medications affecting MRI results.
4. Positional heterogeneity in the liver that could affect imaging quality.
5. History of liver resection or treatment.
6. Inability to coordinate respiration during the MRI scan.

#### 2.2.2 Detecting and classifying focal liver lesions

**Figure 2:**
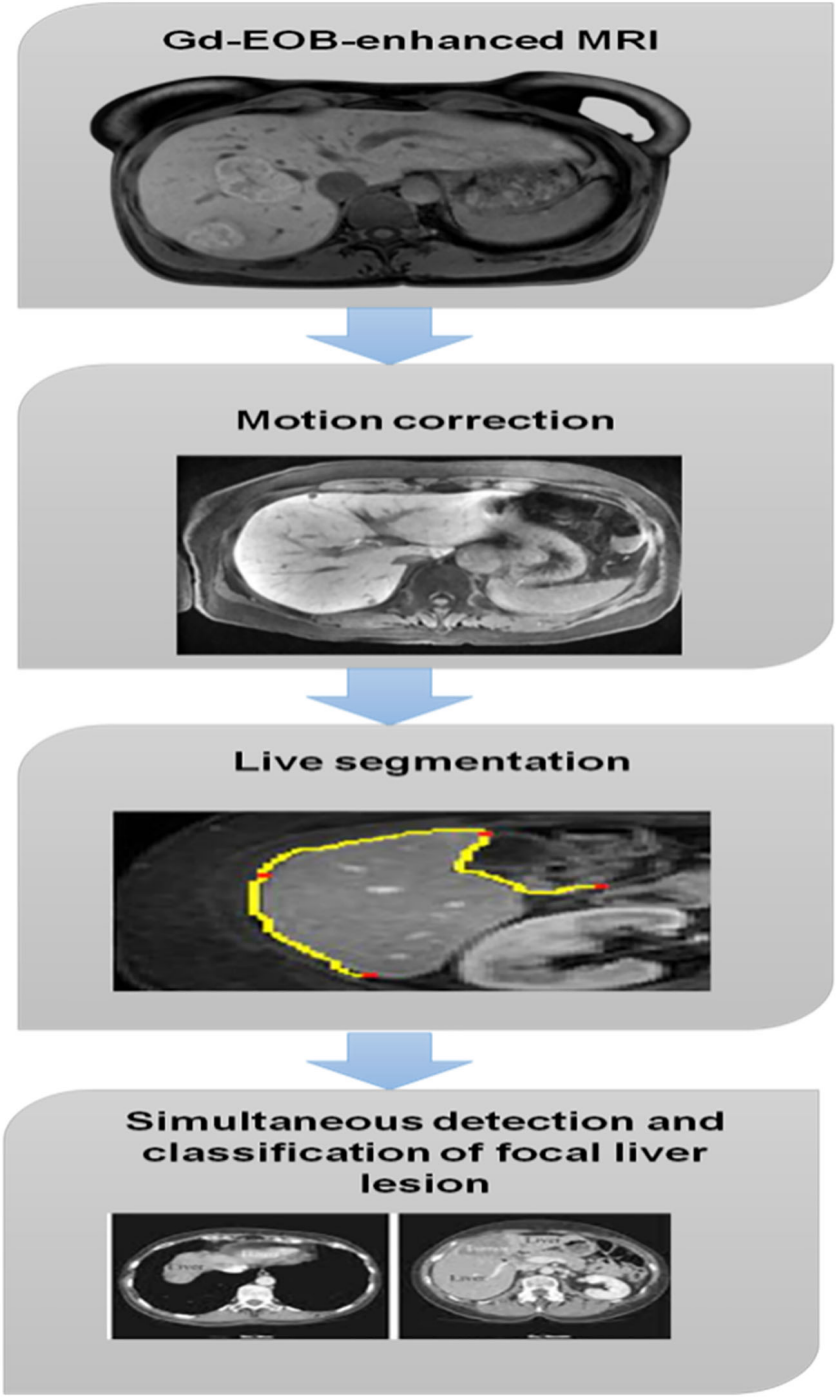
Focal liver lesion.

First, we employed multi-group enrollment to fix the shake in every single image for the whole of our detection and classification procedure. The motion correction method we employed required a parameter file to determine the best possible parameters for registration. Second, we used deep learning to separate the liver tissue. Then, we classified the voxels within each window and looked for signs of a localized liver lesion using our deep learning-based approach. The results from each window were then added together, and the probabilities associated with each voxel’s possible pathologies were determined using a 6-valued probability scale. As a last step, we analyzed how well our algorithm could identify and classify various objects. The new design differed from the old one in that its last layer, instead of being a sigmoid function for classification, was a softmax function. The procedure for identifying and categorizing localized liver lesions is depicted in **Figure 2**.

Using the following formula, we calculated the intermediary field sizes used in (1).


(1) $$r_{intermediate}=\{\begin{array}{c} \frac{r_{out}}{n},m\leq r_{out}\\ 1,r_{out}<m \end{array}$$

where $r_{out}$ is the output kernels length of the convolution layer and m is a fixed numeric value. m is a threshold value. Depending on whether $r_{out}$ is greater than or equal to m , different rules apply for calculating $r_{intermediate}$ . n as a normalizing factor, this variable serves as a divisor or scaling factor when $m\leq r_{out}$ . Its purpose is to scale down $r_{out}$ by a factor of n when the condition $m\leq r_{out}$ is met. The choice of n can depend on various factors such as the desired scale of $r_{intermediate}$ , the nature of the data, or the requirements of subsequent computations.

A liver lesion that had already been segmented slid over the diagnostic window. Input patches used a five-phase configuration of Gd-EOB-DTPA-enhanced MRI. Predicted probability that a given sample was not HCC, a metastasis, a hemangioma, a cyst, a scar, or a negative (normal liver tissue) sample were the network’s outputs. For each voxel in the patch, our network calculates the probability of six different diseases (HCC, metastasis, hemangiomas, cysts, scars, and normal), with the total of these probabilities always equaling 1. In this investigation, a logarithm of the Dice loss suggested in an earlier paper was employed as the loss function. We used a modified Dice loss ( $F_{Dice}$ ) (as Equation 2) that is calculated per patch to train our model.


(2) $$F_{Dice}=-\sum_{f}In\left(\frac{2\sum_{j}\left(x_{j,f}.s_{j,f}+\epsilon \right)}{\sum_{j}\left(x_{j,f}.s_{j,f}+\epsilon \right)}\right)$$

The correlating surface label, $s_{j,f}$ [0, 1] is a small constant value, and $x_{j,f}$ [0, 1] is the output of the lth communication in the last network level. As a starting point, setting $F_{Dice}\;$ to 0 is suggested.

$x_{j,f}$ : This represents the predicted probability of voxel j*j* belonging to class f*f*. It is the output of the model for a specific voxel and class.

$s_{j,f}$ : This is the ground truth label for voxel j*j* and class f*f*. It is a binary value (0 or 1) indicating whether the voxel belongs to the class.

ϵ : A small constant (e.g., 10^−8^) added to the numerator and denominator to avoid division by zero and to ensure numerical stability.

$\sum_{f}$ : The summation over all classes *f*.

In : The natural logarithm function.

#### 2.2.3 Deep learning based on enhanced multi gradient deep convolution neural network

EMGDCNN model is influenced by biology and has great results in medical image analysis. There are various fundamental components included. The convolutional layer is the most important part of the CNN model. The steps of its operation are as follows Equation 3:


(3) $$Z\left(e,p\right)=L\left(\sum_{t=1}^{T}\sum_{p=1}^{r}\sum_{e=1}^{r}y_{e,p,c}\times u_{e,p}^{j}+x^{j}\right)$$

The inputs $\;y_{e,p,c}$ the bias term $x^{j}$ on j , and the convolution kernel $r^{j}$ T are all shown in Equation 3.

Z(e,p) : This is the final output function, which depends on the voxel e*e* and the class p*p*.

L : This is an external function that transforms the result of the inner summation. It could be an activation function (e.g., Sigmoid, ReLU) or a loss function (e.g., Cross-Entropy Loss).

$\sum_{t=1}^{T}$ : This summation is over time steps *t* from 1 to *T*. This suggests that the formula may be considering temporal data.

$\sum_{p=1}^{r}$ : This summation is over classes p*p* from 1 to *r*. This indicates that the formula is aggregating information across different classes.

$\sum_{e=1}^{r}$ : This summation is over voxels *e* from 1 to *r*. This suggests that the formula is aggregating information across different voxels.

$y_{e,p,c}$ : This represents the label or ground truth for voxel *e*, class *p*, and possibly another dimension c*c* (which could be a channel or feature).

$u_{e,p}^{j}$ : This represents a weight or coefficient associated with voxel *e* and class p*p* for the *j*-th feature or channel.

$x^{j}$ : This represents the input feature or channel j*j*.

The Sigmoid functional is the most popular choice for the activating layer’s L input signal:


(4) $$L\left(y\right)={\left(1+\frac{1}{a^{y}}\right)}^{-1}$$

The following steps lead to Equation 4:


(5) $$L^{\prime }\left(y\right)=L\left(y\right)\left[1-L\left(y\right)\right]$$

With a bigger relative value of y in Equation 5, the gradients of the function tend to flatten. The ReLU function is as follows:


(6) $$eLu\left(y\right)=\{\begin{array}{c} y,y>0,\\ 0,y\leq 0. \end{array}$$

**Figure 3:**
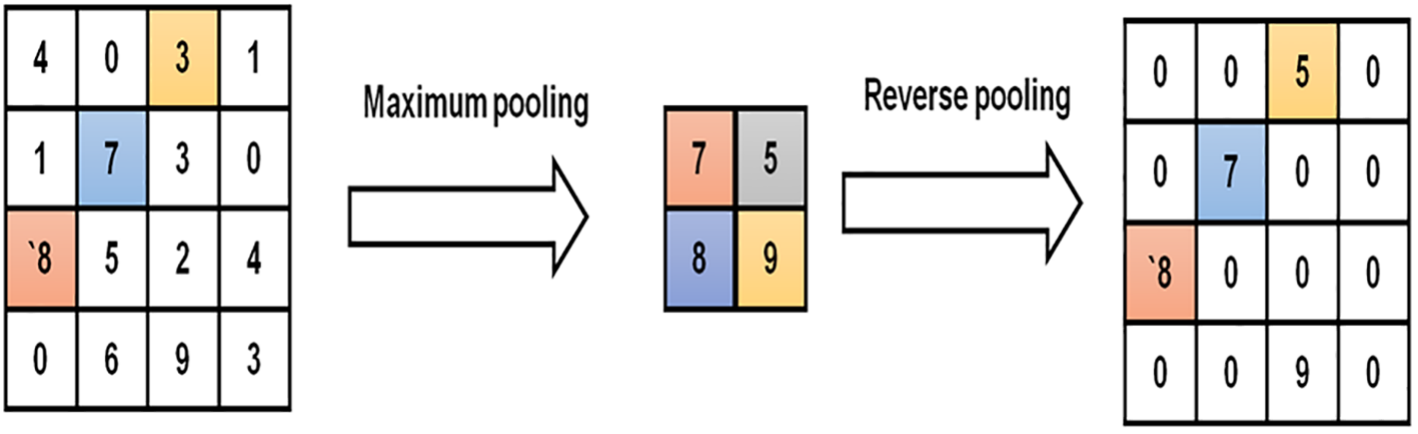
Maximum pooling and deploying.

Besides drastically cutting down on computation time, Equation 4 is also sparse. It is extensively employed in deep convolutional neural networks since it helps prevent performance drops in the network. As a straightforward downsampling process, the pooling layer can help cut down on computation and parameters. Two of the most popular pooling layers are the average pooling layer and the maximum pooling layer. On the other hand, deploying occurs at an upsampling layer. The location of the activation value is recorded if the pooling is at its maximum throughout the network forwarding operation; otherwise, it is set to 0. **Figure 3** depicts the maximum possible pooling and deploying.

The randomized inactivation layer’s purpose is to stop the classifier, which is described as follows:


(7) $$K_{m}^{n}=Bernoulli\;\left(B\right)$$

(8) $${\tilde{X}}_{m}=K_{m}\times X_{m}$$

K is the probability value, ${\tilde{X}}_{m}$ is the initial output, $X_{m}$ is the output following random inactivation, and m is the number of layers in Equations 7 and 8. In this layer, the probability is assigned to an integer between 0 and 1, and each training creates a binary distribution vector. The output is the original integer unless the result is zero, in which case it is 0. This can help to reduce overeating and overfitting. The following is how the Learning algorithm is defined in this study:


(9) $$soft\;max\left(\alpha_{j}\right)=\frac{a^{\alpha_{j}}}{\sum_{r=1}^{E}a^{\alpha_{r}}}$$

In Equation 9, E is the integral gain with pass loss is determined as follows, in which $\alpha_{j}$ is the quantity of the specific problem domain.


(10) $$F\left(O,B\right)=-\sum_{j=1}^{U\times Z}\sum_{r=1}^{E}B_{j,r}\times \text{log}\left(o_{j,e}\right)$$

In Equation 10, it is assumed that o is the final output result, B is the programmed label of the screening test, and $B_{j,r}$ and $o_{j,e}$ , respectively, stand for the gold standard’s label and the first point’s category on the prediction result. These variables only have results of 0 or 1, so they are not included in the equation.

### 2.3 Network architecture and implementation details

#### 2.3.1 Enhanced multi-gradient deep convolutional neural network architecture

The EMGDCNN model was designed to leverage the strengths of deep learning for the detection and classification of localized liver lesions in Gd-EOB-DTPA-enhanced MRI. The architecture consists of multiple convolutional layers, each followed by activation functions, pooling layers, and dropout layers to prevent overfitting. Specifically, the model employs a series of 3x3 convolution kernels, which are effective at capturing spatial features while maintaining a manageable computational cost.

##### 2.3.1.1 Convolutional layers

The input to the network is a 5-phase series of Gd-EOB-DTPA-enhanced MRI images. Each convolutional layer applies a set of filters to the input, generating a feature map. The convolution operation is defined as follows Equation 11:


(11) Output=f(Input∗T+b)

where ∗ denotes the convolution operation, *T* is the convolution kernel, *b* is the bias term, and *f* is the activation function, typically ReLU (Rectified Linear Unit), which introduces non-linearity into the model.

##### 2.3.1.2 Pooling layers

To reduce the spatial dimensions of the feature maps and thus decrease the number of parameters, max-pooling layers are used. Max-pooling retains the most significant features while downsampling the image.

##### 2.3.1.3 Dropout layers

Dropout is a regularization technique where neurons are randomly “dropped out” during training to prevent co-adaptation and improve generalization. The dropout rate is set to 0.5, meaning 50% of the neurons are dropped during each training iteration.

##### 2.3.1.4 Fully connected layers

After several convolutional and pooling operations, the feature maps are flattened and passed through fully connected layers. These layers are responsible for the final classification. The last layer uses a softmax activation function to output the probabilities for each class (HCC, metastasis, hemangioma, cyst, scar, and normal tissue).

#### 2.3.2 Training parameters and implementation

The EMGDCNN was implemented using TensorFlow, a popular open-source machine learning framework. The model was trained on a high-performance computing cluster with NVIDIA GPUs to accelerate the training process.

##### 2.3.2.1 Training data

A total of 132 cases were collected from the Shanghai Public Health Clinical Center. The data were split into training (70%) and testing (30%) sets. Data augmentation techniques such as random rotations, translations, and flips were applied to the training set to increase the diversity of the training samples and improve the model’s robustness.

##### 2.3.2.2 Loss function

The loss function used for training was a modified Dice loss, which is particularly suitable for segmentation tasks. The Dice loss is calculated as follows Equation 12:


(12) $$Dice\;Loss=1-\frac{2\times \sum_{i=1}^{N}p_{i}\times g_{i}}{\sum_{i=1}^{N}p_{i}^{2}+\sum_{i=1}^{N}g_{i}^{2}}$$

where $p_{i}$ and $g_{i}$ represent the predicted and ground truth labels, respectively, and N is the number of voxels.

##### 2.3.2.3 Optimization algorithm

The Adam optimizer was used for training, with a learning rate of 1e-4. The learning rate was reduced by a factor of 0.1 if the validation loss did not improve for 5 consecutive epochs, a strategy known as learning rate decay.

##### 2.3.2.4 Batch size and epochs

The batch size was set to 16, and the model was trained for 100 epochs. Early stopping was employed, and the training was halted if the validation loss did not improve for 10 consecutive epochs.

##### 2.3.2.5 Regularization

L2 regularization was applied to the weights of the fully connected layers to penalize large weights and prevent overfitting. The regularization parameter was set to 1e-4.

## 3 Result analysis and discussion

SPSS was used to perform statistical analysis. To illustrate the radiomic signature’s capacity for prediction, receiver operator characteristic curves were built. Fisher’s exact probability approach was used to evaluate its importance. Prognostic evaluations were made using Kaplan-Meier analyses. The cutoff for significance was P 0.05. In statistical hypothesis testing, the p-value quantifies evidence against the null hypothesis, representing the likelihood of observing the test statistic or more extreme values assuming the null is true. If the p-value falls below a predetermined significance level, usually α=0.05, the null hypothesis is rejected in favor of the alternative, indicating statistical significance. For instance, a p-value of 0.0001 suggests strong evidence against the null hypothesis at the 0.05 significance level.

### 3.1 Fisher’s exact tests

Any sample size may be utilized using Fisher’s exact method, while small samples are where it is most usually applied. Since the importance of departure from an anthropocentric principle may be determined exactly rather than depending on an estimate it became exact in the ending as the sample group expands to infinity, this exact testing method is named after its creator, Ronald Friedman.


(11) $$B=\frac{\frac{s_{1}!}{r_{11!}r_{12!}}\frac{s_{2}!}{r_{21!}r_{22!}}}{\frac{n!}{d_{1!}d_{2!}}}\;$$

There are equation model factors in the numerator and one in the denominator respectively. It takes a computer program to accurately calculate an equation coefficient. The computation of the equation coefficient may allow us to avoid computing three interests for every coefficient.


(12) $$\frac{Q!}{Z!\left(Q-Z\right)!}$$

(13) $$\frac{\left(N\right)\left(N-1\right)\left(N-2\right)\ldots \left(Z+1\right)}{\left(N-Z\right)!}$$

Feldman and Kluge showed that the possibility of each more severe table could be computed using a simple technique that needed no factorials at all after estimating the likelihood of the projected data table. If the lowest frequency in the table is labeled Q and the remaining three frequencies are Z, N, and in clockwise order from Q, then


(14) $$\overset{´}{B}=\frac{r_{a}r_{b}}{r_{e}^{'}r_{f}^{'}}B$$

However, if alternating each frequency is required for the hypothesis under consideration, it may be calculated as follows:


(15) $$B=\frac{r_{e}^{'}r_{f}^{'}}{r_{a}r_{b}}\overset{´}{B}$$

The B of the additional tail is then added to $\overset{´}{B}$ , the possibility that was previously provided for each test.

### 3.2 Results

The proposed model is activated in CAD software for Gd-EOB-DTPA-enhanced MRI, and its efficacy is compared to that of current models like Convolutional Neural Networks (CNN) (22), Support vector machines (SVM) (23), and K-Nearest Neighbors (KNN) (24). We evaluated AUC, specificity, and sensitivity by utilizing all experimental and established methods.

**Figure 4:**
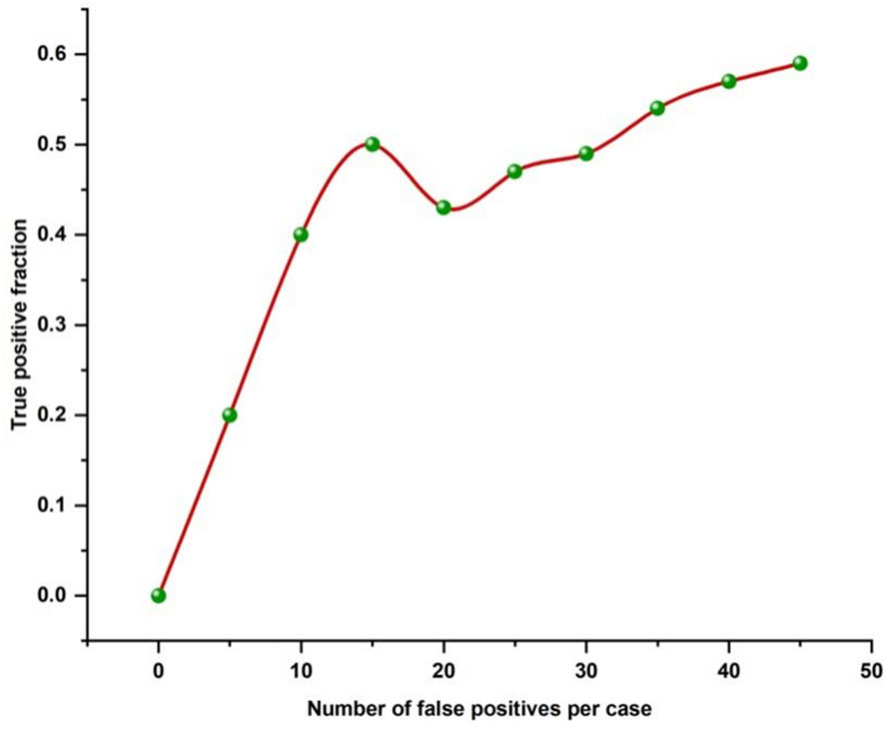
FROC line for all diagnosed localized liver lesions by EMGCNN.


**Figure 4** displays the FROC line for all 40 cases of localized liver lesions found by EMGCNN. The TPR was 0.6 with an average of 25 FPs per case. To measure how accurately lesions were identified, the true-positive ratio (TPR) was computed across all test cases.

(16) $$TPR=\frac{TP}{TP+FN}$$

True positive (TP) and false negative (FN) is abbreviated here. To determine how successfully our proposed technique categorized different types of localized liver lesions, we computed the FROC curve for each output and analyzed the data distribution.

**Figure 5:**
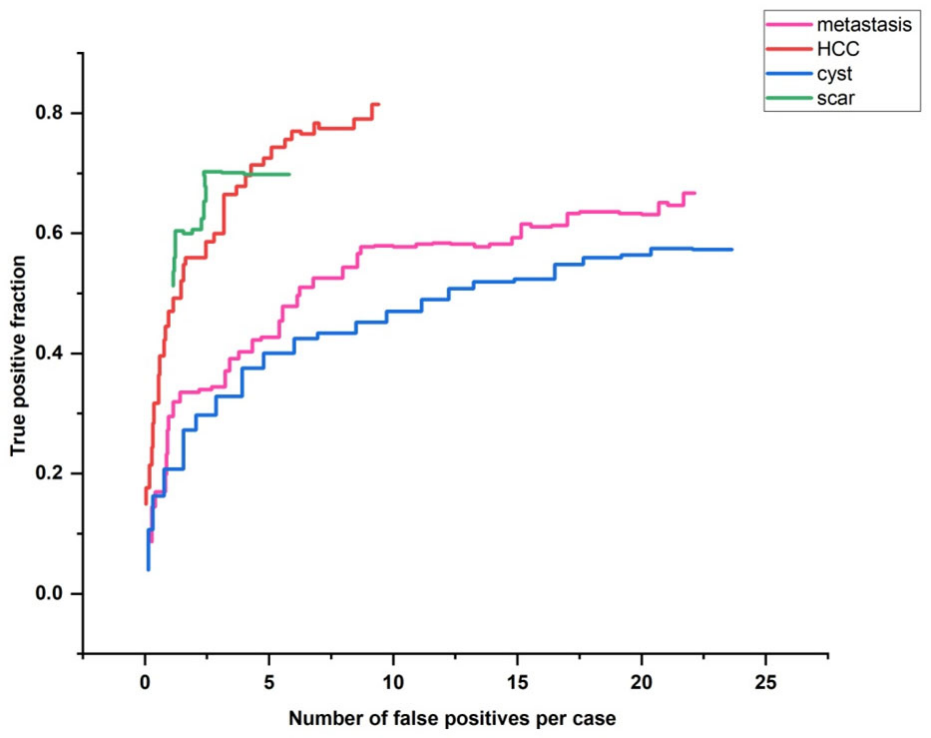
FROC output lines for each type of lesion.

**Figure 5** depicts the FROC output lines for each corresponding lesion type. The direct and obvious revealed an average TPR of 0.56 for HCC, with 8.71 FPs per instance. Overall, the TPR for metastasis was 0.72, with cases averaging 5.00 FPs. The metastatic output channel outperforms the other lines in terms of detectability. Hemangiomas could not be detected by the hemangioma output channel. **Table 2** displays the categorization outcomes for the various lesion types. The accuracy of the categorization was 0.790. The outcome suggests that our suggested strategy might recognize and categorize a localized hepatic lesion.

**Table 2:** FROC output lines for each type of lesion.

Predicted label						
Methods		HCC	Cyst	Scar	Hemangioma	Metastasis
**Actual label**	HCC	27	4	0	0	13
	Scar	0	1	7	0	1
	Cyst	2	135	5	0	3
	Metastasis	13	10	1	0	49
	Hemangioma	0	4	1	0	0

Methods: This column lists the different machine learning or classification methods used to predict the type of liver lesion. HCC (Hepatocellular Carcinoma): The number of cases where the method predicted the lesion as hepatocellular carcinoma. Cyst: The number of cases where the method predicted the lesion as a cyst. Scar: The number of cases where the method predicted the lesion as a scar. Hemangioma: The number of cases where the method predicted the lesion as a hemangioma. Metastasis: The number of cases where the method predicted the lesion as metastasis.

**Figure 6:**
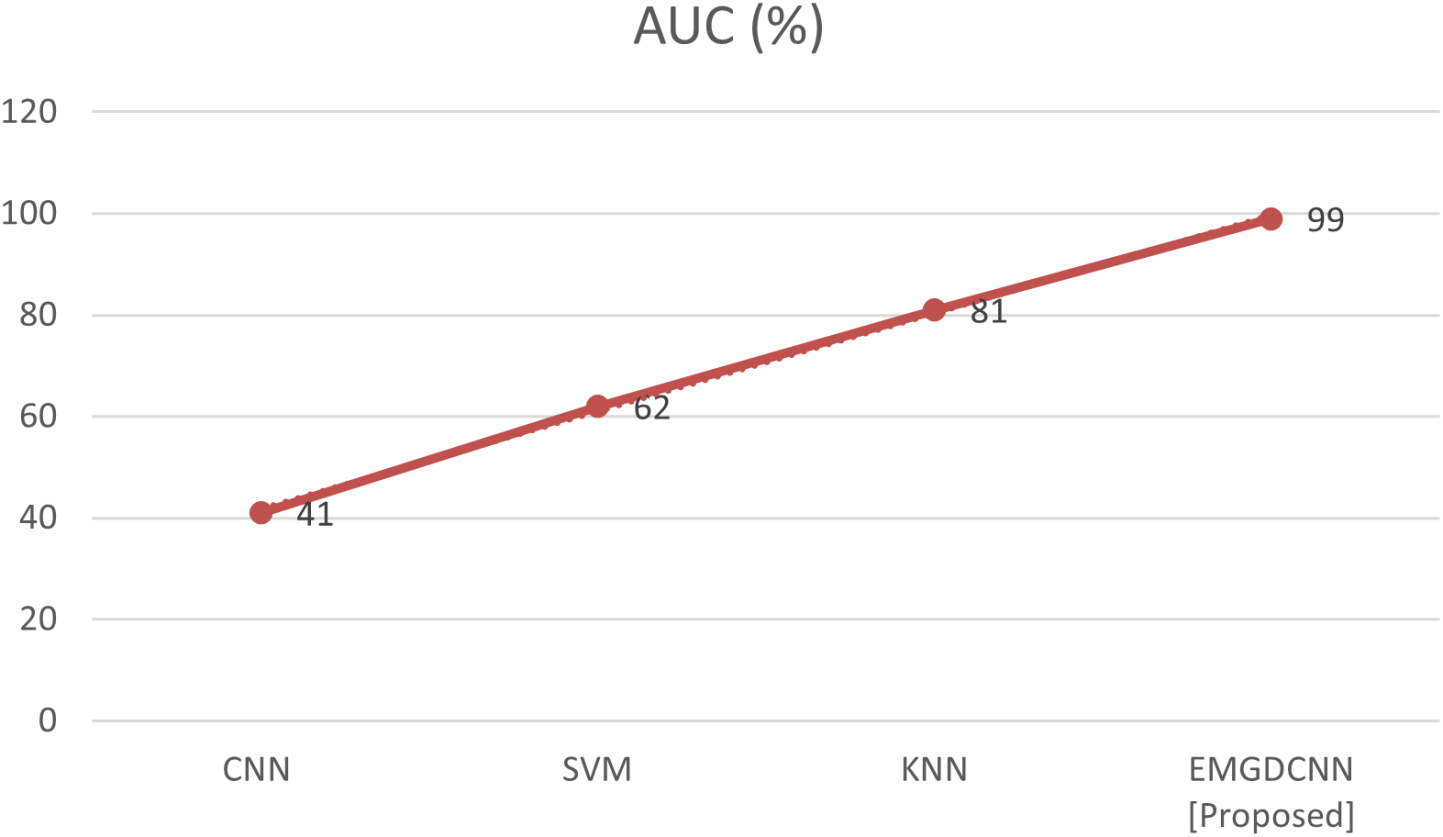
Comparison of AUC.

The area under the curve is shown in **Figure 6**. Calculating the area under the curve to assess the selectivity and measure learning (AUC). It just specifies the distance between things or the scale used to measure them. AUC offers a cumulative evaluation of performance over a wide variety of classifiers because it is independent of size and criterion. The AUC rate shows how well the model distinguishes between positive and negative categories. A higher AUC rate indicates the improved performance of the model. Comparing the [EMGANN] methodology to current methods like CNN, SVM, and KNN reveals a greater level of AUC. The performance of AUC is shown in **Table 3**.

**Table 3:** AUC performance analysis.

	AUC (%)
**CNN**	41
**SVM**	62
**KNN**	81
**EMGDCNN [Proposed]**	90

AUC (Area Under the Curve): A metric used to evaluate the performance of binary classification models. It represents the probability that a classifier will rank a randomly chosen positive instance higher than a randomly chosen negative one. The value ranges from 0 to 100%, where a higher AUC indicates better model performance. CNN (Convolutional Neural Network): A type of deep learning model commonly used for image recognition and classification tasks. SVM (Support Vector Machine): A supervised machine learning algorithm used for classification and regression analysis. KNN (K-Nearest Neighbors): A non-parametric method used for classification and regression, which classifies data points based on their proximity to other points in the feature space. EMGDCNN [Proposed]: The Enhanced Multi-Gradient Deep Convolutional Neural Network proposed in this study, designed specifically for detecting and classifying focal liver lesions in Gd-EOB-DTPA-enhanced MRI images.

The percentage of test samples that are predicted to yield favorable findings from an experiment is referred to as “sensitivity.” It is an accurate portrayal of the case with promising results. The value of sensitivity is calculated using the following equation:


(17) $$\text{Sensitivity}=\frac{TP}{TP+FP}$$

**Figure 7:**
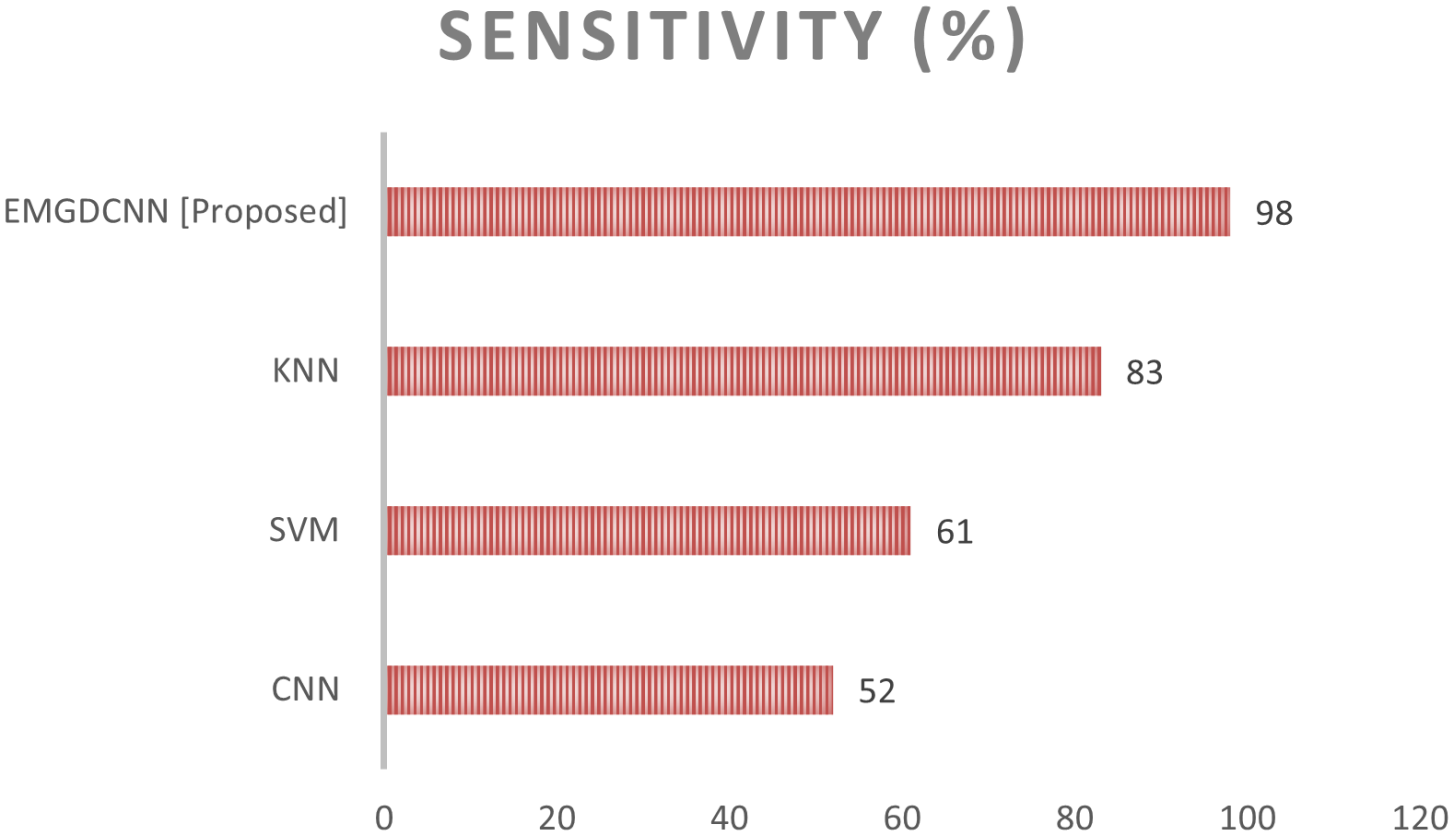
Comparison of the sensitivity.

The comparison of the sensitivity is shown in **Figure 7**. Comparing the [EMGANN] methodology to current techniques like CNN, SVM, and KNN reveals a better degree of sensitivity. The performance of sensitivity is shown in **Table 4**.

**Table 4:** Sensitivity performance analysis.

	Sensitivity (%)
**CNN**	52
**SVM**	61
**KNN**	83
**EMGDCNN [Proposed]**	95

Sensitivity (%): Sensitivity measures the proportion of actual positive cases that are correctly identified by the model. A higher sensitivity indicates that the model is better at identifying true positive cases, reducing the number of false negatives.

The term “specificity” refers to a classifier’s capacity to properly predict the true negatives. This method stands out because it is so accurate at locating Normal instances. This may be stated mathematically as,


(18) $$\text{Specificity}=\frac{TN}{TP+FP}$$

**Figure 8:**
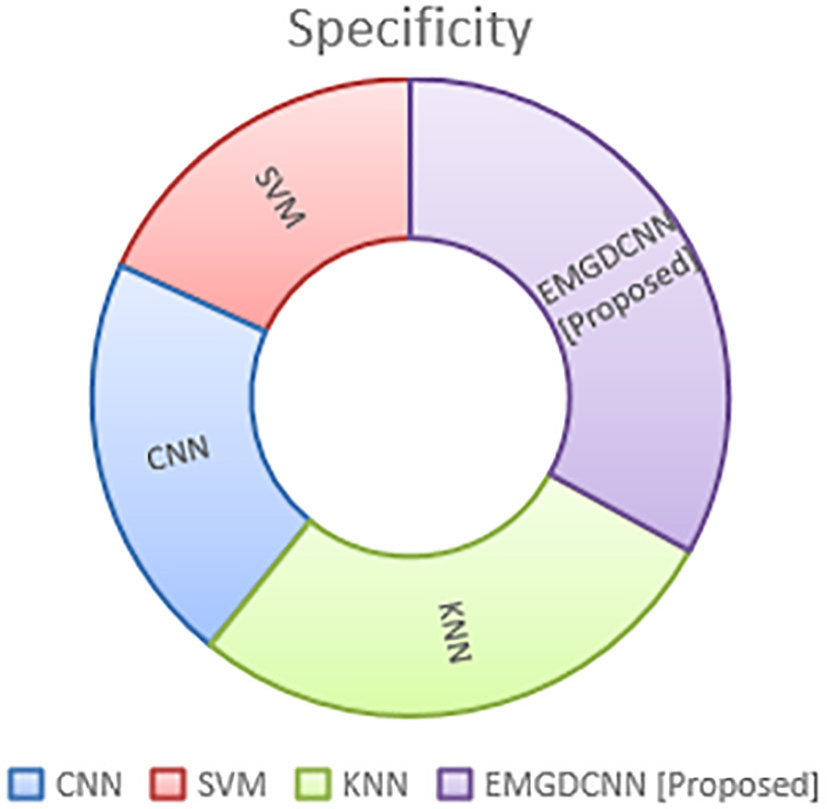
Comparison of the specificity.

**Figure 8** illustrates the specificity’s difference. Compared to currently utilized networks like CNN, SVM, and KNN, the suggested EMGCNN is more efficient. The performance of specificity is shown in **Table 5**.

**Table 5:** Specificity performance analysis.

	Specificity (%)
**CNN**	61
**SVM**	53
**KNN**	81
**EMGDCNN [Proposed]**	96

Specificity (%): Specificity measures the proportion of actual negative cases that are correctly identified by the model. A higher specificity indicates that the model is better at identifying true negative cases, reducing the number of false positives.

### 3.3 Discussion

#### 3.3.1 Limitation and future work

The article acknowledges the relatively high false positive rate of 25-26 per case, which can lead to unnecessary follow-up tests and increased patient anxiety. However, the research justifies the Enhanced Multi-Gradient Deep Convolutional Neural Network (EMGDCNN) model based on its overall strong performance in detecting and classifying liver lesions. The model achieves a true-positive ratio (TPR) for full focal liver lesion detection, with an AUC of 90%, sensitivity of 95%, and specificity of 96%. These metrics indicate that the EMGDCNN is highly effective at distinguishing between different types of localized liver lesions, which is crucial for accurate diagnosis. Specifically, the TPR for metastasis is 0.72, highlighting the model’s strength in detecting this type of lesion. The high AUC, sensitivity, and specificity suggest that the model outperforms other methods like CNN, SVM, and KNN. While the high false positive rate is a limitation, the model’s ability to both detect and classify lesions simultaneously, along with its overall diagnostic accuracy, makes it a valuable tool for radiologists. Future work could focus on refining the model to reduce false positives while maintaining its high detection and classification capabilities.

Additionally, the research does not discuss the model’s robustness across different clinical settings. This is an important consideration, as the performance of the model may vary depending on the specific characteristics of the patient population, imaging equipment, and clinical protocols. To address this, future studies should evaluate the EMGDCNN in diverse clinical environments, including different hospitals, with varying levels of equipment, and among patients with a wide range of liver conditions. This would help to ensure that the model remains effective and reliable in real-world clinical practice.

To ensure the generalizability and robustness of the EMGDCNN model, it is crucial to validate its performance on an external dataset. An external validation dataset would consist of Gd-EOB-DTPA-enhanced MRI scans from a different source or population, providing a more rigorous evaluation of the model’s performance in real-world clinical scenarios. Future studies should aim to include an external validation step, ideally from multiple centers, to assess the model’s reliability across various imaging conditions and patient demographics.

Including an external validation dataset will help to confirm that the EMGDCNN remains effective and reliable when applied to diverse patient populations and imaging environments. This will be a critical step in establishing the model’s utility as a diagnostic tool for radiologists.

#### 3.3.2 Comparison with State-of-the-Art Methods and Recent Relevant Works

In this study, compared the performance of our Enhanced Multi-Gradient Deep Convolutional Neural Network (EMGDCNN) with several established methods, including Convolutional Neural Networks (CNNs), Support Vector Machines (SVMs), and K-Nearest Neighbors (KNN). The results showed that the EMGDCNN outperformed these methods, achieving an AUC of 90%, sensitivity of 95%, and specificity of 96%.

However, to further validate the effectiveness of the EMGDCNN, it is essential to compare it with more state-of-the-art approaches. For instance, recent studies have explored the use of advanced deep learning architectures such as U-Net (25), DenseNet (26), and Residual Networks (ResNets) (27) for medical image analysis, which have shown promising results in various imaging modalities, including MRI. These networks incorporate sophisticated features like skip connections, dense blocks, and residual blocks, which can potentially improve the model’s ability to capture complex patterns and features in medical images.

Additionally, there has been significant progress in the development of ensemble methods, where multiple models are combined to improve overall performance. Techniques such as bagging, boosting, and stacking have been applied to medical image analysis, often leading to improved accuracy and robustness (28). Incorporating such ensemble methods could be a valuable future direction for enhancing the EMGDCNN.

Furthermore, recent work has focused on integrating attention mechanisms into deep learning models, which allow the network to focus on the most relevant parts of the image. Attention-based models, such as those using self-attention or transformer architectures, have demonstrated superior performance in various computer vision tasks, including medical image segmentation and classification (29).

Another emerging trend is the use of generative adversarial networks (GANs) for data augmentation and synthetic data generation, which can help address the issue of limited training data in medical imaging (30). GANs can generate realistic synthetic images that can be used to augment the training dataset, potentially improving the generalization capabilities of the model.

Recent relevant works also highlight the importance of incorporating multi-modal information and temporal dynamics in the analysis of liver lesions. For example, studies have shown that combining MRI with other imaging modalities, such as ultrasound or CT, can lead to better diagnostic outcomes (31). Additionally, the analysis of dynamic contrast-enhanced (DCE) MRI sequences, which capture the temporal changes in tissue enhancement, has been shown to improve the detection and characterization of liver lesions (32, 33).

In conclusion, while the EMGDCNN has demonstrated strong performance in detecting and classifying focal liver lesions, it is important to benchmark it against more state-of-the-art methods and to consider the integration of recent advancements in deep learning. Future work should explore the potential benefits of incorporating advanced architectures, ensemble methods, attention mechanisms, and multi-modal data. By doing so, we can further enhance the model’s performance and ensure its relevance in the rapidly evolving field of medical image analysis.

## 4 Conclusion

In this research, we proposed using an EMGDCNN to detect and categorize a focal liver lesion on Gd-EOB-DTPA-enhanced MR scans. One hundred thirty-two cases were collected from the Shanghai Public Health Clinical Center in China. As expected, our method was able to identify and categorize specific liver lesions. Test cases showed a true-positive ratio of 0.5 for detecting full focal liver lesions, with a total of 26 false positives per case. AUC, sensitivity, and specificity were some of the metrics tested in this experiment. An EMGDCNN was proposed, and its results were 90% of AUC, 95% of sensitivity, and 96% specificity. The suggested method performs better than the existing methods. According to the findings of our research, it is conceivable to use a single network for both detection and classification at the same time. To be of assistance to radiologists, it is required to significantly increase detection capability.

## Data Availability

The original contributions presented in the study are included in the article/supplementary material. Further inquiries can be directed to the corresponding author.
